# A Reexamination of the Relationship between Training Practices and Welfare in the Management of Ambassador Animals

**DOI:** 10.3390/ani14050736

**Published:** 2024-02-27

**Authors:** Steve Martin, Grey Stafford, David S. Miller

**Affiliations:** 1 Natural Encounters, Inc., 127 Conservation Way, Winter Haven, FL 33884, USA; 2 Adjunct Faculty, College of Natural Sciences, Grand Canyon University, 3300 West Camelback Road, Phoenix, AZ 85017, USA; 3 Miller Veterinary Services, PLLC, P.O. Box 2786, Loveland, CO 80539-2786, USA; davemillerdaczm@gmail.com

**Keywords:** ambassador animal, control, choice, reinforcement, punishment, institutional support, training, staff development, enrichment

## Abstract

**Simple Summary:**

Contemporary best practices for working with exhibit animals in zoological facilities incorporate behavioral research, theories on operant learning, animal welfare principles, and practical experience to provide animals with largely positive experiences when interacting with their caretakers and their environment. These best practices must be more fully implemented to increase ambassador animal welfare based on taxonomic and individual animal characteristics, experience with present and past caretakers, and ambassador program objectives. This implementation requires training staff to modify animal behavior using the most positive and least intrusive interactions possible. We rigorously consider how to apply best practices to meet ambassador program objectives and animal welfare needs by ensuring appropriate institutional support, the selection of the most suitable animals for the program’s objectives, and the creation of an environment where animals recognize that they can choose whether to interact with humans and participate in programs, as well as exert control over their environment. Our synthesis provides a framework showing how staff can continually assess and address the behavioral response of ambassador animals to their environment—along with their interactions with humans during and outside of program usage—with the goal of meeting our most current and evolved criteria for ensuring high animal welfare.

**Abstract:**

There is an ethical need to document and develop best practices for meeting ambassador animals’ welfare needs within the context of meeting zoo and aquarium program objectives. This is because ambassador animals experience direct and frequent contact with humans. This paper rigorously synthesizes behavioral research and theory, contemporary practices, and personal experiences to offer key concepts that can be applied to meet ambassador animal welfare needs. These key concepts include addressing an animal’s recognition of choice and control, the use of the most positive and least intrusive effective interventions when training animals to participate in programming, and an overall reduction in aversive strategy use. Our model for increasing ambassador animal welfare focuses on seven main areas of concern, including the following: choosing the most suitable animal for the program; choosing the human with the right skills and knowledge for the program; using the most positive, least intrusive, effective training methods; developing a strong trusting relationship between trainer and animal; developing a comprehensive enrichment program; the need for institutional support; and creating opportunities for animals to practice species-appropriate behaviors. Our model will provide guidelines for improved ambassador animal welfare that can be refined with future research.

## 1. Introduction

Modern zoos evolved from menageries, carnivals, and circuses [1] where animals were confined behind bars or glass. Animals were initially perceived as objects to view and guests were attracted to exhibitions by the opportunity for close encounters with unusual and charismatic species. These facilities subsequently evolved beyond being strictly entertainment-oriented into educational institutions that attracted a cross-section of the populace to marvel at the diversity of the animal world [2]. Patrons wanted to know more about the animals they viewed and zoo administrators responded by developing educational programs that supported zoos with additional visitation and income [1]. They formed education departments and the number of animal species featured in educational programs increased. Education programming was further codified by becoming a standard addition to mission statements and later became a requirement for trade association accreditations [3,4,5,6]. Zoos became valued by the public as a place for families to discover new things together and learn more about the natural world [7].

As education programs flourished, public, professional, and scientific concerns for the welfare of animals in zoos and aquariums increased in the early 2000s [8]. This attention to animal welfare inspired many articles and research projects focused on factors believed to have a direct effect on zoo animal welfare [8,9,10]. Animal welfare activities influenced most areas of zoo operations, such as exhibit design, animal accession and disposition, health care, behavioral training and management methods, nutrition, and natural and veterinary-assisted breeding. Trade associations came to regard welfare as a component of conservation [3,4,5,6,11]. The Association of Zoos and Aquariums (AZA) Accreditation Standards clearly describe the requirement for a formal process of assessing animal welfare and the quality of life for animals in human care [3]. These standards also include descriptions of the value of ambassador animals in audience engagement, delivering educational messages, enrichment, and planning. However, there is little mention in these standards of how ambassador animals should be trained to participate in programs, even though inappropriate training methods are associated with significant negative impacts on animal welfare [12,13,14,15,16,17,18,19,20]. Furthermore, there is a dearth of published empirical research or theory that can inform best practices for specifically meeting the welfare needs of ambassador animals being handled in programs.

Education, conservation, research, and recreation are important operation objectives found in many zoos’ mission statements [3,4,5,6,21,22]. Animal welfare supports each of those target activities, and should therefore be a key mission objective for all zoological facilities. Educational programs at zoological facilities involve a wide variety of species and guest experiences, with target audiences ranging from preschoolers to adults.

These programs most often present animals in close proximity to visitors on facility grounds. However, offsite programs are also conducted at schools, churches, hospitals, fairs, and other public attractions. Given the growth in the use of live animals in educational programs, and considering their unique ability to facilitate connections between visitors and their wild counterparts, these animals are now referred to as ambassadors. Ambassador animals can be presented inside or outside of their enclosure, generally with the intent of providing visitors with close or direct contact. Direct contact may include physical contact, feeding, or other interpretive experiences such as touch tanks [3,4,5,6].

This paper synthesizes behavioral research, theories on positive and negative reinforcement, animal welfare principles, and practical experience in a framework that enables staff to rigorously evaluate how training best practices can be applied to benefit ambassador animal welfare. Our synthesis (1) examines the relationship between ambassador animal training, enrichment, husbandry, staff training, institutional support, handling, and the wellbeing subsequently experienced by those animals; (2) addresses facility management and physical structure, which includes zoological parks, aquariums, aviaries, and various other facilities that house ambassador animals, which we will henceforth refer to as zoos; and (3) provides a model to assist staff when assessing how to best meet animal welfare needs by making recommendations that are based on our combined 60+ years of experience with ambassador animal programs, selection of the “right” ambassador animals, and considerations that are applicable to the humans who work with ambassador animals, such as their approach to animal handling and training. Our intent is to provide guidelines and a cognitive framework that will elevate ambassador animal welfare and stimulate research that will result in further refinements of our model.

## 2. Defining Welfare

A variety of definitions have been developed for animal welfare, all of which concern animals’ physical, emotional, and overall state of mind and health [23]. Alternatively, some definitions distinguish between wellbeing—what the animal is experiencing and how it is responding to external stimuli—and welfare, which focuses on the external management in place to achieve wellbeing [24]. While acknowledging these potential differences, we use the term welfare with the understanding that the focus is on achieving optimal animal wellbeing in whatever manner is defined for a specific individual of a certain species in a given setting. This is often conceptualized on a linear continuum but can also be considered a multidimensional concern that considers multiple factors, including but not limited to physiology, behavior, ecology, and health. An animal’s behavior provides valuable insights into health and wellbeing, and can be a crucial indicator of welfare status.

The Association of Zoos and Aquariums Animal Welfare Committee defines welfare as follows: an animal’s collective physical, mental, and emotional states over a period of time. This measured on a continuum from good to poor. An animal typically experiences good welfare when it is healthy, comfortable, well-nourished, and safe, is able to develop and express species-typical relationships, behaviors, and cognitive abilities, and is not suffering from unpleasant states such as pain, fear, or distress. Because physical, mental, and emotional states may depend on one another and can vary from day to day, it is important to consider these states in combination with one another over time to provide an assessment of an animal’s overall welfare status [25]. Definitions can be seen in Table 1 below.

## 3. Moving toward a More Positive Future

The movement away from forceful strategies towards positive-reinforcement-based management methods incorporates the concept of providing animals with choice and control, buzzwords frequently aligned with good welfare. While often used as if they are one word or concept (i.e., giving more choice and control = improved welfare) the terms are distinct. Choice is the animal’s ability to choose between multiple relatively salient behavioral options while control refers to an animal’s ability to produce desired results, such as producing an expected effect [12]. Individuals exercise control over the environment by making choices [13]. The more choices and options that are available in a particular situation, the greater the degree of behavioral freedom of choice [31]. Freedom can also be considered as a condition in which there is no aversive control of behavior [32]. Control is critical for healthy development, and conditions of a diminished sense of control can lead to declining physical health, a high frequency of stereotypic behavior, maladaptive behaviors, and reduced wellbeing [13,17]. Belief in one’s ability to exert control over the environment and produce desired results is essential for an individual’s general wellbeing [13].

These concepts of degrees of freedom, control and choice are important for animal care professionals to understand and utilize when training ambassador animals with positive reinforcement. However, using positive reinforcement to train animals does not, in itself, always promote good welfare. In training environments where the only way to access a valuable reinforcer is to perform a specific target behavior, the action can be considered coercive [17,31,33]. To provide a high degree of freedom of choice when training animals with positive reinforcement, the process should include at least one alternative choice to the target behavior for which an animal can gain reinforcement. The more choices, the more degrees of freedom the animal has [16].

Goldiamond describes freedom as the availability of alternative responses, as well as the reduction in coercive conditions through the increase in available alternative responses [16]. Goldiamond also describes genuine choice, where behavioral alternatives denote the availability of alternative contingencies that are equally possible [31,33]. For genuine choice to be available to an animal, the alternative behaviors and reinforcers need to be equally valuable and attainable. For instance, when teaching a parrot to step from a perch onto a person’s hand for a peanut reinforcer, an alternative behavior, such as walking a short distance down the perch to earn a similar peanut reinforcer, would be available. It is important to note that Goldiamond also describes freedom as a matter of degrees, and implies coercion is a matter of degrees as well [31,33]. Based on our decades of experience training ambassador animals, genuine choice is not always practical for all training situations. However, animal care professionals striving to enhance the welfare of their animals can implement training plans and strategies designed with the consistent goal of moving toward the genuine choice end of the freedom continuum.

It is becoming more common for trainers to provide multiple opportunities for animals to choose between various behaviors when training a target behavior [17]. For instance, walking with an ambassador African Crested Porcupine *(Hystrix cristata)* on a leash limits the animal’s choices and control by restricting its speed, direction, and the distance it can walk from the handler. Taking the leash off the porcupine gives it more control so it can choose to navigate its environment in any manner it wants. It can choose to walk by the handler’s side and gain the occasional treat as a reinforcer for doing so or it can choose to walk away and pursue other salient reinforcers, such as the smell of another animal’s urine on a bush, as well as digging in the ground to explore an interesting smell, or climbing on a fallen tree. It is not always safe to remove the leash and give all ambassador animals complete control, for instance, when walking an animal near a busy road or past other animals. However, a person can reduce the intrusiveness of the leash, and provide a higher degree of freedom, by providing multiple opportunities for the porcupine to earn reinforcers while walking on the leash close to the trainer’s side so the leash is slack and not putting pressure on the animal.

The same concept is true for other ambassador animals when restraint is required to keep an animal safe from running off, jumping out of a presenter’s hands, or, in the case of a bird, attempting to fly off a trainer’s hand. In these situations, a trainer can use the differential reinforcement of alternative behavior (DRA) [34] to teach the animal to perform behaviors that replace the undesirable behavior. Some examples of alternative behaviors with a raptor might include reinforcing the behaviors of the bird turning its head to one side, lifting a foot. Associated with the DRA strategy is the differential reinforcement of incompatible behavior (DRI) [34], where the target behavior is mutually exclusive to the unwanted behavior, i.e., the animal cannot perform both behaviors at one time, such as reinforcing the duration of the behavior of a hawk sitting on the glove or leaning its wing against the trainer’s arm. The hawk cannot perform either of those behaviors and fly off the glove at the same time. A mammal can learn to sit calmly on a stump, table, or mat on the floor, while in the presenter’s arms. A mammal can learn to perform multiple behaviors that are incompatible with the behavior of jumping out of the person’s hands, such as holding on to a person’s shirt or arm, leaning against a person’s chest, or targeting its nose to a person’s hand. All of these behaviors have multiple contingencies for reinforcement that give the animals choices that increase their control over their environment, and degree of freedom, while earning reinforcers. The goal is to keep animals safe from harm by providing a wide variety of choices for behaviors that the animal can engage in during the time it is restrained by the presenter.

Control is also a reinforcer for behavior [17]. Teaching an ambassador animal, such as a striped skunk (*Mephitis mephitis),* to enter a crate and receive a reinforcer after the door closes is a common training strategy. However, if the closed door denies the skunk access to the outside, the reinforcer’s influence on future behavior may not be strong enough to increase the likelihood of the skunk entering the crate the next time the opportunity occurs. Closing the door with the animal inside may even punish (reduce) the future behavior of going inside.

A trainer can give the skunk control in the following scenario:

- The skunk enters the crate and receives a food reinforcer after the door closes.
- The trainer opens the door when the skunk moves toward it, allowing the animal to leave the crate.

The skunk can control the door’s opening by moving toward it. Control acts as a reinforcer for the behavior of entering the crate, and the future frequency of entering the crate increases. Repeatedly giving the animal control of the door might influence it to ultimately stay inside the crate to receive additional reinforcers even when the door is opened [35]. The next step is to increase the duration of calm behavior in the crate as the trainer moves the crate.

Aversive management methods—negative reinforcement and positive punishment—can mislead a handler into believing their coercive strategies are successful because effective punishment can reinforce the punisher [20]. Research shows capture and handling can be stressful for most ambassador animals, including reptiles [18]. Studies have shown that aversive training methods negatively affect animal welfare and that there is no evidence that aversive training methods are more effective than reward-based training [14].

The high-pressure environment caused by the need to be delivered on time to presentation areas can create conditions where even the most compassionate animal caregivers might momentarily abandon their better judgement and reach in to pick up an animal against its will. When escape behaviors prove ineffective, some animals may submit to the aversive stimulus, allowing themselves to be picked up. This can mislead handlers to believing the animal likes being held because of its apparently placid behavior. What handlers mistake as calm behavior can actually be the result of flooding the animal with aversive stimuli, resulting in learned helplessness [26], which is mentioned in Table 1: a condition in which an animal learns that no matter what it does it cannot escape an aversive stimulus and subsequently gives up trying. The handler’s focus on successfully getting the animal to the educational program can blind them to realizing the value of—or the opportunity associated with—teaching animals to voluntarily participate in the activity. The handler’s coercive behavior is reinforced by the success of getting the animal in hand, while the animal’s escape behavior is punished. This management process perpetuates forceful animal handling at the expense of animal welfare and can lead to compromised relationships between humans and animals [18].

Some ambassadors participate in programs while free of restraints, such as in free-flight bird shows. The absence of restraints gives animals a wider variety of choice, including the choice of whether to participate in the program or not. However, removing restraints does not innately give free-flighted birds more choice and control. What if a bird refuses to perform the target behavior, or flies away? In this case, it is possible for a trainer to believe that reducing diet and weight is a way to motivate the animal to perform more reliably. However, there are many influences on motivation that do not involve hunger, such as relationship with the trainer, current antecedent conditions, physical ability to perform the behavior, and past history of punishment or reinforcement with the behavior [35]. Training free-flighted birds and other animals working free of restraints without an over-reliance on using hunger as a motivator requires exceptional knowledge and skill on the part of the trainer. The challenge for modern zoos and aquariums is to empower ambassador animal handlers with the skills, knowledge, and decision-making authority to maximize the use of the most positive, least intrusive, effective strategies for behavior change while also improving welfare. Success in this area can promote desirable behavior, improve welfare, and enhance guests’ knowledge and satisfaction.

## 4. The Effects of Stress and Distress

Organisms have evolved a range of adaptive cellular, systemic, and behavioral stress responses to physical, environmental, biological, and/or psychological stressors. Neuroendocrine responses to stressors include increases in corticosteroids in the General Adaptation Syndrome model, which is the process an animal’s body goes through when it is exposed to any kind of stress, positive or negative [27]. These corticosteroids are often measured in zoo animals as a part of assessing animal responses to various stressors [36]. When an animal is presented with stressors under infrequent, acute conditions that it can cope with, they often prompt short-lived physiological and/or behavioral outcomes that are conducive to the animal’s survival (eustress in the General Adaptation Syndrome model) [27,37,38].

A strategy for improving animals’ perceptions of stressors is to provide animals with options for choice and control [39,40]. However, when stressors like eliminating an organism’s choice and control over its environment are presented chronically, whether in terms of frequency or duration, those same adaptations can result in maladaptive, inappropriate, long-term behavioral and/or physiological outcomes (distress in the General Adaptation Syndrome model) [27,41,42]. These outcomes can include negative impacts on indices of animal welfare, such as animal’s fecundity, parental care, homeostasis, resilience, lifespan and other functions [43]. While further research is needed regarding the impacts of training methods on animal welfare, the preceding suggests that an array of detrimental effects may result from trainers using conventional practices where the chronic application of positive punishment occurs, or choice and control are otherwise eliminated from an animal’s purview during handling and other interactions. To promote the improved welfare of animals in training programs, ambassador animal handlers and trainers should have a plan in place to systematically reduce, and ultimately eliminate, training practices that involve positive punishment and negative reinforcement.

## 5. A Model for Improved Ambassador Animal Welfare

Taking a holistic view of ambassador animal programs with the goal of improving the welfare of the animals who participate, we focus on the following seven main areas of concern:

- Choosing the right animal for the program;
- Choosing the right human for the program;
- Using the most positive, least intrusive, effective training methods;
- Developing a strong trusting relationship between trainer and animal;
- Developing a comprehensive enrichment program;
- Institutional support;
- Designing the public program to create opportunities for animals to practice species-appropriate behaviors.

### 5.1. Choosing the Right Animal for the Program

There is a need to carefully consider the characteristics of an individual animal, the available facilities, and a program’s objectives when selecting an animal for an ambassador program (Table 2). Before acquiring an animal, research should be conducted to understand the past history of the animal, including their training, health and physical condition, temperament, and other behavioral traits. It is recommended that a video of the animal, including handling and training experience, be received from the holding facility to help in the evaluation process and the training process before the animal is acquired. The ambassador animal’s health status should be thoroughly assessed before it joins an ambassador animal program. This health status should also be evaluated regularly as part of the continued assessment of suitability for inclusion in an education program. For example, data collected over a 30-year period showed that flight-impaired raptors with injuries that classify them as non-releasable were often associated with chronic, progressive medical conditions that negatively affected the future welfare of those birds [44]. Fractures near joints, such as in the wrist, elbow, or shoulder, often lead to chronic osteoarthritis and other degenerative joint diseases undetectable by observation and even physical exams because birds tend to hide their painful symptoms [44]. Daily behavior observation and a yearly diagnostic evaluation, including radiographs, should be part of every non-releasable raptor’s health and welfare evaluation. This is an excellent practice for geriatric or otherwise medically compromised ambassador animals of any species.

Having a plan in place to monitor and manage an animal’s wellness is only part of the operative procedures required to promote optimal animal welfare. The plan should also include monitoring each animal’s behavior challenges, training progress, weight fluctuations, participation in programs, interactions with trainers and guests, and any milestone experiences, such as giving birth, biting or aggressive incidents, flyoffs or escape experiences, etc. Also, this plan should include options for housing if and when an animal is deemed unfit for inclusion in ambassador animal programs [45]. Perhaps the most important step is for management and keeper staff to open a dialog with veterinarians about the difficulties associated with observing and quantifying pain in ambassador animals and their ability and responsibility to perform routine quality-of-life assessments. Having this dialog already established will help navigate conversations about the difficult, but often necessary, decision to resolve chronic pain through compassionate euthanasia [44].

### 5.2. Choosing the Right Human for the Program

At the heart of any comprehensive training program are the humans who create, guide, and implement the plan. Because zoological facilities work to balance education with welfare in an environment of restrictive budgets, volunteers and docents are often viewed as practical alternatives to hiring full-time staff members. However, many docents and volunteers work only a few days a month, which is often an insufficient amount of time to establish the consistency required to build trusting relationships with animals and gain training skills foundational for programs that prioritize optimal animal welfare. Zoological facilities looking to improve ambassador animals’ welfare face a challenge far more complicated than asking whether it is best for volunteers or full-time staff to handle and train animals. The most important questions should be “Does this particular person have the skills required to create a trusting relationship, and use the most positive, least intrusive form of training with this particular animal in these conditions and, if not, how do we fulfill our program obligations while enhancing and improving the welfare of the animals?”

Although full-time schedules improve consistency, much learning and experience is required to effectively implement the most positive, least intrusive strategies when working with any animal. Expert training begins with understanding operant conditioning and behavior change principles, and continues with accurately applying those principles for the animal in training. Knowing the science provides a good foundation, with the recognition that some individuals will be more adept at understanding and applying the training concepts than others. The goal should be for all personnel working with ambassador animals to participate in ongoing education to refine their knowledge and training skills so as to achieve greater consistency [18]. The ultimate goal should be to create an institutional memory in training competency, where every person working with ambassador animals can apply their skills equally to every animal in the collection. It is important that observable handling and training skills be the primary consideration when evaluating staff members for promotions to positions that require contact with ambassador animals. This should be more important than more commonly used factors, such as seniority.

### 5.3. Using the Most Positive, Least Intrusive Training Methods

Alberto and Troutman [46] described three important behavior change principles: 1. If a less intrusive procedure will accomplish the desired behavior change, it is neither necessary nor ethical to use a more intrusive procedure. 2. If the choice is between a less intrusive but ineffective procedure and a more aversive but effective procedure, then the effective procedure should be selected. 3. Before a more intrusive procedure is employed, data should be collected to substantiate the ineffectiveness of the less intrusive procedure. Drawing from Alberto and Troutman’s work, Friedman created the Hierarchy of Behavior-Change Procedures, based on the least intrusive principle, to help animal trainers evaluate their training procedures and encourage the most positive, least intrusive, effective strategies for any given situation [20].

Friedman’s hierarchy of least-to-most intrusive behavior change principles is a cautionary tool that encourages trainers to slow down and consider the intrusiveness of each level of interaction with an animal before moving to the next level of the procedure for changing behavior. Level 1 of the hierarchy suggests that an animal’s health, nutrition, and physical abilities be evaluated before moving toward training interventions. Level 2 recommends arranging antecedent conditions to promote desirable behavior. Level 3 encourages the use of positive reinforcement to shape or encourage desirable behavior. Level 4 of the hierarchy encourages trainers to proceed with caution and consider the previous strategies before moving to the use of DRA strategies to replace unwanted behavior. Level 5 includes extinction, negative punishment, and negative reinforcement. Level 6 is positive punishment. With both level 5 and level 6, caution and a reevaluation of the previous plan and actions should be used when making the decision to use these advanced procedures. Friedman suggests that the majority of behavior problems can be solved with strategies found in levels 1–4, and, under some conditions, procedures in level 5 may be ethical and effective choices. Level 6, positive punishment, is rarely necessary, or only recommended when one has requisite behavior knowledge and teaching skills.

Research has shown that the use of aversive training methods (positive punishment and negative reinforcement) can jeopardize the physical and mental health of animals [14], and cause escape behavior, aggression, phobia, and apathy [20,47]. Additionally, Friedman points out the following problems with punishment: 1. Punishment does not teach learners what to do instead of the problem behavior. 2. Punishment does not teach caregivers how to teach alternative behaviors. 3. Punishment is really two aversive events—the onset of a punishing stimulus and the forfeiture of the reinforcer that has maintained the problem behavior in the past. 4. Punishment requires an increase in aversive stimulation to maintain initial levels of behavior reduction. 5. Effective punishment reinforces the punisher, who is, therefore, more likely to punish again in the future, even when antecedent arrangements and positive reinforcement would be equally, or more, effective. Punishment is not more effective than using positive reinforcement when training animals [18].

An outstanding advancement in animal welfare is growing through the use of positive reinforcement by zoological staff to teach animals to voluntarily participate in critical medical and husbandry behaviors [17,18,48]. Animals are now learning to voluntarily participate in full body inspections, injections, blood draws, and hoof trims, while avoiding the experiences associated with being restrained, darted, and anesthetized for these necessary procedures [18]. This is relevant to ambassador animals because these animals have traditionally been presented while being held by an interpreter who restrains the animal in some fashion. The use of manual/handling restraints limits the level of control an animal has in its environment, and thus poses the possibility of reducing the animal’s welfare by limiting the animal’s option for choice and even creating the potential for injury for some species, especially non-raptorial birds with fragile legs that may be susceptible to injury when jesses are used, such as corvids, kookaburras, and tawny frogmouths [49].

Flight-impaired birds are often used as ambassadors in educational programs. When a raptor with a wing injury attempts to fly off the glove (called bating), there is a significant possibility of causing further damage to the injured wing as well as causing increased stress and reduced welfare. To avoid the aversive effects of negative reinforcement and punishment, a sensitive and skilled trainer can evaluate an animal’s subtle body language behavior, make adjustments to the antecedent conditions, and significantly reduce the bating behavior. As mentioned above, a trainer can also use differential reinforcement of incompatible behavior to replace bating behavior with a more desirable behavior, such as sitting calmly on the gloved hand of a trainer.

As contemporary animal care professionals learn more about problem-solving and the science of behavior change principles, one of the most important lessons they should learn is to ask empowering questions. It may be natural for some trainers to focus on what they dislike about a certain situation and try to build a plan to stop the problem behavior. However, rather than asking how to stop an undesirable behavior, a more useful approach is asking what behavior is desired. The answer to this question often leads a trainer to a positive reinforcement strategy of the differential reinforcement of incompatible behavior (DRI) [34].

Many trainers have difficulty understanding precisely when punishment from the trainer or the environment is in play. They may also lack awareness of the potential problems that can arise from using punishment. A common example of punishment seen in some ambassador animal programs involves walking an ambassador animal on a leash. When the animal begins pulling ahead or puts significant pressure on the leash, the handler’s response may be to tug back on the lead to punish (i.e., reduce) the animal’s behavior of pulling ahead. This may result in the animal reducing the pulling behavior and temporarily walking next to the trainer. However, after a few moments, the animal is likely to pull ahead again. The trainer responds by tugging back on the leash with more force to make more of an impression in another attempt to reduce the behavior. This scenario will likely repeat many times during the walk and may involve uncomfortable, even dangerous, levels of escalation of the aversive stimulus.

Key takeaways from this punishment scenario are as follows: (a) the aversive stimulus, pulling back on the leash, needs to increase for it to have an effect on the behavior due to the animal’s habituation to the aversive stimulus [20]; (b) the temporary reduction in the animal’s pulling behavior reinforces the human’s behavior of tugging back on the leash, making them more likely to tug again in the future [20]; and (c) studies have shown that low- or medium-level punishers will not suppress a behavior over time [19]. This punishment scenario plays out time and again as handlers attempt to control ambassador animals as they try to jump out of a person’s hands, fly off a glove, slither away on a table, crawl up a person’s arm, or otherwise escape being held.

The alternative to using punishment when working with ambassador animals is to train the animals to voluntarily participate in programs while free of restraints. Although this is possible for some trainers, facilities, and situations, it is not always advisable, given the level of training skill found in typical ambassador animal programs at present. Keeping animals and humans safe from harm is a goal for every ambassador animal program, and restraints can be one way to keep animals safe.

However, too often, people accept punishment-based behavior because they do not realize that punishment is in play at these times. As an example, people might say things like, “the bird is going to bate” and “the serval is going to tug on the leash”. To improve welfare with an animal on restraints, a more positive, less intrusive, effective method is often found in the way trainers use restraints with ambassador animals. Teaching people to use jesses, harnesses, leashes, and other restraint devises as safety tools instead of as training tools should be a minimum requirement for all ambassador animal programs.

Following Friedman’s hierarchy, using the differential reinforcement of incompatible behavior to teach animals what to do rather than what not to do should apply to all ambassador animals, whether they are skunks, servals, owls, porcupines, or any other animals in an education program where restraints are in use [20]. A “loose leash” goal should be part of every training plan for ambassador animals where restraints are used. It is worth saying again: restraints should be safety tools, not training tools.

### 5.4. Developing a Strong Trust Account between Trainer and Animal

The construct of trust can be operationalized, as the level of certainty in interactions will result in good outcomes, such that future interaction increases [50]. Trust can be seen in an animal’s approach behavior to a known individual, and a lack of trust can be seen in an animal’s escape behavior, or even very subtle body language showing concern at the approach of an unknown individual. Zoo animals’ wellbeing will increase through a bond of trust that can be significantly strengthened through training [48]. Human–animal relationships are formed though repeated, consistent, and positive interactions [51], and are often specific to individual people, who animals discriminate from others [52]. Every trainer has a trust account with every animal they work with [35,50,53]. When they have a positive interaction, such as offering a food reward or providing opportunities for control, they make a deposit into that trust account. A key consideration that is sometimes overlooked is correctly interpreting the body language of the animals in a person’s care, and then giving each animal a voice through their body language. Even the tiniest look of an eye, movement of hair, or rustle of feathers can signal to an astute trainer when to move forward, stop, or back away. When caregivers respond to these subtle signals in ways that make an animal more comfortable, as determined by its body language, they give the animal more choice, more control, and a vote in deciding what the caregiver should do in a particular situation. Having multiple opportunities to make choices and have more control over the contingencies in their lives gives animals a higher degree of freedom [31] and improved welfare [12,13,16,17].

Withdrawals from the trust account come from aversive interactions like force, threats, and punishment [50]. The goal of a trainer should be to make enough deposits that the relationship can withstand the occasional withdrawal that might be associated with scenarios such as pulling back on a leash to keep an animal from moving into a dangerous situation, or medical exams or emergencies that require temporarily restraining an animal for the animal’s safety. Through the use of positive reinforcement training techniques, animal caregivers create trusting relationships with animals. This benefits the caregivers, who pursue programmatic, husbandry, and medical behavior training goals, and the animals, which have reduced stress and improved welfare [18].

Although the focus is often on trust between humans and animals, trust also relates to the way animals perceive and respond to environmental conditions [35]. For instance, animals build trust in transport-crate training through the repetition of positive reinforcement for entering and exiting a crate. Trust can be built through repeated interactions with novel objects, going through doorways, and stepping onto a perch or other station. If an animal perceives that their approach will result in positive outcomes, the approach behavior increases. When behavior that was trained in a quiet environment with few distractions is repeated in novel environments with more distractions, the animal builds trust with its trainer, as long as that exposure is paired with repeated positive experiences. The building of trust is evident in situations where an animal’s approach behavior replaces cautious, tentative, or escape behavior.

Trust accounts can be bankrupted by actions like forcing animals to comply with commands where aversive stimuli are consequences for non-compliance, restraining animals, moving too quickly through doorways, or forcing animals toward novel objects and situations when their body language shows that escape behavior would occur without the restraint [50]. These aversive actions compromise the human–animal relationship and lead to the animal’s approach behavior being replaced with escape behavior [15]. At this point, there is the potential for some trainers to blame the animal and wash their hands of responsibility for the undesirable behavior. This is often when derogatory labels are used to blame the animal for their poor performance when the problem is the conditions the trainer created for the session. Expert trainers understand that an animal’s behavior reflects their expertise, and they accept responsibility for undesirable behavior, which, in turn, motivates them to seek more productive interpretations of the animal’s behavior and build positive reinforcement training plans that result in better training outcomes and improve animal welfare [54].

### 5.5. Developing a Comprehensive Enrichment Program

Enrichment is often considered to be additions made to an animal’s environment with the intention of promoting physical and psychological wellbeing [18]. However, there is much more to a comprehensive enrichment program than adding scents and novel items to an animal’s enclosure. Life for an animal in human care should be filled with opportunities to learn new associations and contingencies in ways similar to their wild counterparts [55]. Young describes enrichment as divided into five non-mutually exclusive categories:

- Social (i.e., social grouping);
- Occupational or cognitive (e.g., opportunities for mental or physical exercise);
- Physical (e.g., the use of species-appropriate furniture in enclosures);
- Sensory (i.e., stimulation of the five senses);
- Nutritional (i.e., the use of food, associated or not with devices that enable animals to use their anatomical and behavioral adaptive features in food handling) [55].

Enrichment programs should be goal-based and evaluated against their intended purpose [56]. Vicino explains the process of developing an Outcome-Based Husbandry (OBH) program that shifts the focus of enrichment from an input-based program to one that enhances an animal’s ability to use its skills and relevant behaviors to engage with their environment [56]. Enrichment is not limited to the holding areas for ambassador animals. It extends to the transport and even the area where the animals are presented in enrichment programs. When ambassador animals voluntarily participate in programs to earn reinforcers, the activity is enriching [48,57]. Training can provide animals with the motivation, skill, and confidence to make the most successful use of the enrichment opportunities that are provided [48]. Considering the use of training as a form of enrichment, Westlund suggests the following four criteria by which an intervention may be considered enrichment: 1. the enrichment should give the animal more control over its environment; 2. enrichment should increase the animal’s behavioral choices; 3. enrichment should promote species-appropriate repertoires; and 4. Enrichment should empower the animal to deal adequately with challenges. She demonstrates that formal training using operant conditioning fulfills all these criteria [57]. Operant conditioning, with a focus on positive reinforcement, provides a framework for successful enrichment practices [58], and is itself enriching because it is mentally and physically stimulating and creates an environment where animals have choice and control [57]. Comprehensive enrichment programs also include a description of goals, the formal planning and approval process, implementation protocols, records of activities, and evaluation and readjustment of the program [8].

Finally, in 2001, Mellen and MacPhee proposed a set of goals that served as a framework for enrichment programs. This framework has come to be known as S.P.I.D.E.R. and still holds up as a valuable guide for enrichment programs for all animals in zoos [59]. The framework consists of setting goals, planning, implementation, documentation, evaluation, and readjustment. Enrichment programs should promote species-appropriate behavioral opportunities, be evaluated each time they are offered, and be further evaluated and adjusted on a regular basis [3].

### 5.6. Institutional Support

Institutional administrators and supervisors are uniquely associated with the welfare of ambassador animals through their support of ambassador animal programs. The AZA Working Group on the presentation of animals states that hall staff assigned to handle animals during presentations must be trained in compliance with the institution’s written animal handling protocols [3]. All staff who work with animals should receive adequate levels of training in the science of animal learning, training, and handling, and continued professional development should be a prerequisite for anyone working with animals [18]. Zoo leaders can help improve ambassador animal welfare by supporting the selection of appropriate species and individuals within those species, supporting housing and transport conditions for ambassador animals, supporting the training of staff members, supporting long-term care for animals that are not appropriate for use in ambassador animal programs, and other initiatives that promote enrichment and improvements in animals’ perceived control over their environment. Training ambassador animals at the highest level to voluntarily participate in programs takes the right animal, the right human, and the most positive, least intrusive training strategy. All of this relies on consistent support from administrators and supervisors to prioritize these elements to create an environment where ambassador animals can experience the highest level of welfare.

### 5.7. Designing the Public Program to Create Opportunities for Animals to Practice Species-Appropriate Behaviors

For ambassador animal programs to pursue education and conservation goals while meeting animals’ welfare needs, long natural history lectures should be replaced with more interactive programs that involve animals performing species-appropriate behavior [60]. Teaching animals to do what nature built them to do is both empowering for the trainers, as they develop trusting relationships with the animals and improve their positive reinforcement training skills, and enriching for the animals, as they gain more control over their environment and reinforcers [18].

Teaching a skunk to use its olfactory sense to locate a piece of food in one small cardboard packet among 20 identical cardboard packets, teaching a porcupine to climb a five-foot-high tree stump for the treat at the top, or teaching an owl to use its extraordinary hearing to locate a very quiet cricket sound playing from a phone hidden behind one of many obstacles are all examples of empowering animals to use their senses and adaptations to earn reinforcers in ways representative of their wild counterparts. Training animals with positive reinforcement to perform species-appropriate behavior in ambassador animal programs has far-reaching benefits for the staff, guests, and most of all, the animals, which receive more choice, more control, and improved welfare [18].

## 6. The New Model in Application: Bird, Mammal and Reptile Examples

### 6.1. Choosing the Right Animal for the Program (Table 3, Table 4 and Table 5)

#### 6.1.1. Birds

Raptors are often used in ambassador animal programs and many, if not most, of these birds have come through rehabilitation programs. Owls that were parent-reared in the wild and come through rehabilitation facilities into ambassador animal programs make exceptionally poor subjects for ambassador animal programs (see “Important Considerations About Parent-reared Owls” below). Fortunately, non-owl raptors who have been injured in the wild and come through rehabilitation programs adjust to life in human care much more easily than owls. Other common birds in ambassador animal programs include tawny frogmouths, kookaburras, corvids, and parrots. Most birds are presented in programs while restrained in some manner, such as with jesses or, in the case of parrots, flight-restricted by clipping their wings. However, it is becoming more common to see free-flighted birds in ambassador animal programs.

**Table 3:** Birds commonly used in ambassador animal programs and examples of key points to consider [61].

Bird	Rationale	Possible Concerns
Red-tailed Hawk (*Buteo jamaicensis*)	Important conservation message; can be calm and voluntary participants with the right training, even after rehab.	Previously injured hawks can have significant medical issues later in life [44].
Laughing Kookaburra (*Dacelo* *novaeguineae*)	Very interesting bird that can be stimulated to reproduce its iconic call; can be trained to fly free by expert trainers.	Legs are not as strong as raptors so caution is needed if using jesses [49]; caution is advised if weight-managing.
Tawny Frogmouth (*Podargus strigoides*)	Calm, almost placid behavior and cryptic coloration that can be demonstrated when sitting on a tree.	Legs are not as strong and stress-resistant as raptors. Caution is advised when using jesses [49], (pp. 12–13, [62]). Watch for straight up posture, and/or cackling noise and fluffed feathers, which may indicate stress.
Blue and Yellow Macaw (*Ara* *ararauna*)	Large, bright, and often colorful with a good story to tell about why they do not make good pets.	Can be very loud when they learn to vocalize for attention. Hand-reared birds that were clipped in their first year but have full wings in subsequent yeas do not know how to control flight well and are at high risk of flyoffs. Free-flight is an expert-only activity.

**Table 4:** Mammals commonly used in ambassador animal programs and examples of key points to consider [61].

Animal	Rationale	Possible Concerns
Striped Skunk (*Mephitis mephitis*)	Good story of scavengers; can be easy to handle when hand-raised and trained properly.	Can get aggressive if restrained against its will; can become overweight; nails may need to be trimmed.
Nine-banded Armadillo (*Dasypus novemcinctus*)	Interesting scavenger and sense of smell. Can be exhibited in pen, where they can run around looking for treats.	Can struggle to adjust to food as a positive reinforcement; handlers should teach them to voluntarily come out of holding area instead of picking up bird while balled up.
African-crested Porcupine (*Hystrix cristata*)	Dramatic appearance; can climb tree stump for program; can be taught to dig; good sense of smell.	Can back into people with quills if upset; can chew out of crates and dig deep burrows; may need to give browse or bones to chew on to keep teeth from over-growing.
Virginia Opossum (*Didelphis virginiana*)	Common nocturnal animal; when hand-raised, is easy to work with for most people; can show natural behavior of carrying leaves and paper in its tail.	Short-lived; can become overweight; may bite; potential for zoonotic disease; therefore, public contact is discouraged.

**Table 5:** Reptiles commonly used in ambassador animal programs and examples of key points to consider [61].

Animal	Rationale	Possible Concerns
African spurred tortoise (*Geochelone sulcata*)	When small, can be excellent program animals; can be target-trained.	May be prone to respiratory infections if they are kept cool in wet enclosures; they get too large to handle, and they are active diggers and climbers; if nutrition is not managed properly, this can cause shell deformities.
Ball Python (*Python regius*)	Generally docile and easy to handle; can be used to convey messages about pets and not to release invasive species.	May have difficulty shedding; may have mites if bought from pet store; can become large and heavy.
Savannah Monitor (*Varanus exanthematicus*)	Large and impressive; great sense of smell; can walk on harness.	Can become overweight without exercise; they get very large and can outgrow their habitat; can live from 10 to 15 years; sharp claws can scratch.
Veiled Chameleon (*Chamaeleo calyptratus*)	Very interesting eating behavior; can take crickets from guests’ hands.	Do not generally recognize water bowls, so misting the enclosure twice a day is advisable; limited handling of up to 20 min with experienced handlers.

**Important Considerations About Parent-reared Owls:**

Owls are some of the most popular ambassador animal species participating in educational programs at present. However, owls that have come through rehabilitation with injuries are the one ambassador animal species that experiences compromised welfare more than any other animal in zoos [60], (pp. 5–6, [62]), [63]. For that reason, they are of special concern in this paper.

Well-meaning zoological professionals often acquire previously injured owls from rehabilitation centers, sometimes with the misconception that they are “rescuing” the bird from an uncomfortable life at the facility. However, owls raised by their parents and subsequently injured in the wild experience severe difficulties adjusting to life in human care [63]. The IAATE lists these core challenges when including parent-reared owls in ambassador animal programs:

- Parent-reared owls do not adjust as well as their human-reared counterparts in terms of the necessary or desired husbandry, medical, and program behaviors.
- Parent-reared owls tend to persistently show behaviors that may indicate a welfare concern (examples include bating off the glove, concealment posture/sitting tall with feathers slicked, beak clacking, hissing, flying away from or at trainers, ducking and flinching, flaring wings, and raising hackles).
- Because parent-reared owls generally exhibit a high rate of escape behavior, training is most often accomplished through flooding with aversive stimuli, (which has a high likelihood of resulting in learned helplessness,) or improper attempts at counter conditioning the fear response, which may lead to an unhealthy weight reduction.
- Parent-reared owls rarely adapt well to life in human care, based on an IAATE report [63]. Parent-reared owls rarely, if ever, voluntarily choose to participate in educational programs. Professional bird trainers on the board of IAATE with over 180 years’ collective experience training owls stated that they would not attempt to train another parent-reared owl because of the welfare compromises that may be involved for the bird [64]. In contrast, the trainers that were interviewed supported the statement that owls raised by humans are likely to voluntarily approach a trainer rather than move away, can readily learn to sit on a gloved-hand, and will even fly to an experienced trainer [63].

The question, then, is what to do with owls that are currently part of ambassador animal programs, do not voluntarily participate in programs, and exhibit signs of stress when handled by humans. For trainers seeking to improve the welfare of parent-reared owls that do not voluntarily participate in ambassador programs, a good option is to place them in an aviary where they are restraint-free and have more choices and control in their environment [64]. Trainers may still be able to work with the birds by placing food through the wire mesh and onto a platform to increase the owls’ approach behavior. Because exhibit owls no longer wear jesses and the trainers do not handle the birds, an owl’s escape behavior may decrease and, after some time, it is possible that calm body language and even confident approach behavior toward staff will occur.

#### 6.1.2. Mammals

A wide variety of mammals participate in ambassador animal programs, from mice to cheetahs, although most ambassador animal programs at zoos avoid including large cats and primates in their collections. We will focus this section on a few of the most common species of mammals found in ambassador programs for use as an example that may be applied to other species. As the animals listed below are all nocturnal in nature, it is important to consider their welfare when training them to participate in diurnal activities. Many zoos have committed to teaching their ambassador animals to voluntarily participate in programs and are even teaching them to participate without restraints. A number of facilities have taught their African-crested porcupines (*Hystrix cristata)* to walk by the trainers’ sides from their holding areas to their presentation areas (personal observation, 9 June 2022, 15 May 2022). Skunks, armadillos, guinea pigs, and many other species of mammals are now voluntarily loading into crates and participating in programs (personal observation, 8 June 2021).

#### 6.1.3. Reptiles

The cognitive abilities of reptiles and amphibians have traditionally been overlooked, resulting in an absence of documented training and enrichment protocols compared to the literature that exists for training mammals and birds [65,66]. It is possible that many people underestimate the learning capabilities of herpetofauna, but it is important to remember that they follow the same laws of behavior as all other animals on this planet and can certainly learn to perform behaviors for reinforcers. Indigo snakes (*Drymarchon couperi)* have been taught to press a key to receive water, and several species of snakes have been taught to shift off exhibit or into buckets on cue [66]. The keepers at one zoo taught their panther chameleon (*Furcifer pardalis*) to walk out of its enclosure onto a stick for transportation to a transport container (personal observation, 13 November 2023), and keepers at another zoo taught a fire-bellied newt (*Cynops orientalis)* to swim into the keeper’s hand to be transported to a feeder bin while the keeper serviced its aquarium (personal observation, 26 January 2021). Reptiles are possibly smarter than many people ever thought, and, for that reason, well-suited to ambassador animal programs.

### 6.2. Choosing the Right Human for the Training Program

The field is moving toward ambassador animals working free of restraints, which includes free-flight birds. The need for skill and experience increases dramatically when attempting to work with animals that are free of restraints in programs performed in uncontrolled environments, such as outdoors, or in large rooms. Teaching an animal to work free of restraints, especially free-flight birds, requires expert level trainers to be accomplished successfully while providing the highest welfare for the animals. The IAATE Position Statement on Free Flight recommends that free-flight trainers should demonstrate the following:

1. A working knowledge of the science of behavior change principles, especially positive reinforcement strategies.
2. The commitment and ability to develop a training program based primarily on the most positive, least intrusive methods, avoiding aversive training strategies whenever possible.
3. A comprehensive understanding of food management and weight management and their ethical application, as described in the IAATE Food Management and Weight Management Position Statement.
4. An awareness of environmental factors that pose a potential safety risk or might reduce motivation.
5. The ability to arrange the environment to set the bird up for success.
6. Knowledge of the natural and individual history of the program birds and the ability to evaluate an individual bird’s suitability for the program.
7. The ability to interpret a bird’s body language and adjust the program or training plan accordingly.
8. The ability to effectively use telemetry.
9. Safe and species-appropriate creance use.
10. The ability to execute the facility’s fly-off protocol [67].

### 6.3. Using the Most Positive, Least Intrusive, Effective Training Methods

For each staff member in an ambassador animal program, using the most positive, least intrusive effective training method should be a baseline commitment. This includes each interaction a handler has with an animal, from acquiring the animal from its home enclosure to presenting the animal in programs, to each step associated with returning the animal to its home enclosure. If animals are presented with any type of restraint device, such as jesses or leashes, there should be a plan in place to ensure each staff member uses the most positive, least intrusive, effective methods of training. These training methods include creating environments where animals have high levels of behavioral freedom by providing multiple ways to earn reinforcers any time an animal is in training. These principles, along with the proficient use of positive reinforcement, should apply to all animals involved with ambassador animal programs, with the goal of improving ambassador animal welfare [27].

### 6.4. Developing a Strong Trusting Relationship between Trainer and Animal

With every animal in an ambassador animal program, trusting relationship are established through the repetition of positive experiences [50,51,53]. Trainers, handlers and other caregivers should always be aware of the body language of the animals in their care and strive to create trusting relationships, which lead to better training outcomes and improved welfare [48].

### 6.5. Comprehensive Enrichment Program (Table 6, Table 7 and Table 8)

It is important to design enrichment programs to fit the needs and special characteristics of each animal. Many ambassador animals are nocturnal by nature (owls, skunks, opossums, porcupines, and armadillos, as an example); therefore, it can be difficult to evaluate the interactions they have with enrichment devices. However, most ambassador animals adjust to a more diurnal schedule, making it easier to observe the animal and collect data through personal observation.

Most raptors spend the majority of their time after eating resting on a perch. They are not inclined to play or explore their environment like a parrot or corvid might be. For that reason, a valuable enrichment condition that a facility can provide for raptors is a good view of the surrounding area and access to the elements. Sun, wind, and rain can all be reinforcers for raptors in specific conditions. A large bath pan and varying sized perches can also add effective enrichment experiences.

Careful consideration should be taken anytime novel items, especially items associated with food, are given to a raptor, especially an owl. For instance, hiding treats in a paper bag or carboard box is a common form of enrichment for parrots, corvids, and many mammals. However, there is an increased possibility an owl will eat the paper product along with the food items. Therefore, owls should be monitored for the ingestion of foreign objects provided as enrichment [68]. More than most other animals, owls are likely to become obsessed with enrichment items where food is hidden or associated and can aggressively protect the item for hours or even days at a time. Browse approved for animals to eat can provide safe enrichment opportunities for many animals, but novel and unnatural objects can also be dangerous for many animals, especially if they are of a size the animal can eat.

**Table 6:** Birds commonly used in ambassador animal programs and examples of enrichment items and possible concerns [61].

Bird	Enrichment Opportunities	Possible Concerns
Red-tailed Hawk (*Buteo jamaicensis*)	A good view overlooking a large area is advisable. Rotating perches, bath pans, opportunities to sit in the elements, the addition of browse, and some items the bird can tear up are acceptable.	Caution should be taken to ensure hawks do not eat enrichment items and are not left in the heat or cold without shelter. Observe the bottom of their feet to look for bumblefoot if perches are not occassionally rotated.
Laughing Kookaburra (*Dacelo novaeguineae*)	Bath pans, changing perches, misters, opportunities to spend time outside in the elements, food wrapped in paper, the scatter feeding of crickets or mealworms, and training are enriching.	Be aware of the possible ingestion of paper and other enrichment items. Be aware of eating insects that may enter the housing area, as they may carry parasites.
Tawny Frogmouth (*Podargus strigoides*)	Calm, almost placid behavior, and cryptic coloration that can be demonstrated when sitting on a tree.	Legs are not as strong and stress-resistant as raptors. Caution is advised when using jesses [49], (pp. 12–13, [62]).
Blue and Yellow Macaw (*Ara ararauna*)	Benefits can be acquired from lots of enrichment items and opportunities, such as browse, misted water a few times a week, and hiding treats in toys or paper items. Training can be enriching.	Care should be taken that bands on legs do not get caught in wire or enrichment items or that birds do not get wrapped up or tangled in rope, such as sisal rope.

**Table 7:** Mammals commonly used in ambassador animal programs and examples of enrichment items and possible concerns [61].

Animal	Enrichment Opportunities	Possible Concerns
Striped Skunk (*Mephitis mephitis*)	Foraging and digging for hidden food are enriching; can learn to be harnessed and taken for walks; training is enriching,	If trained for harness, be sure to give the skunk control and avoid restraining the skunk to put on the harness because they can bite when restrained.
Nine-banded Armadillo (*Dasypus novemcinctus*)	Changing substrates and housing can be enriching, as well as hiding food items that the armadillo has to smell to find, and providing a water tub for them to swim in.	Watch out for wearing on the feet if enrichment encourages digging.
African-crested Porcupine (*Hystrix cristata*)	Digging opportunities, chewing or smelling for an item, wood to chew on, and training are enriching.	Be careful that teeth do not get overgrown from a lack of things to chew; be careful they do not ingest paper where food might be hidden.
Virginia Opossum (*Didelphis virginiana*)	Leaves or paper to gather in their tail for nesting material, digging, scent location, and climbing opportunities are enriching.	Overfeeding of enrichment food items can lead to obesity.

**Table 8:** Reptiles commonly used in ambassador animal programs and examples of enrichment opportunities and possible concerns [61].

Animal	Enrichment Opportunities	Possible Concerns
African Spurred Tortoise (*Geochelone sulcata*)	Boulders or small logs for climbing, items they can climb on or manipulate, softballs, and PVC pipes are enriching.	Will eat most plants it comes in contact with; burrowing is an enrichment opportunity but challenging at times.
Ball Python (*Python regius*)	Scents like new plants, branch clippings and leaves, new hides, branches, tunnels to explore, and handline can be enriching.	Excessive hides or gripping points may become a challenge for programming if the snake is not trained to voluntarily leave its enclosure.
Savannah Monitor (*Varanus exanthematicus*)	Hiding food for the monitor to locate, soaking water bowls, rotating tree branches and substrates, a heated rock for basking, and walking outside on harness training can be enriching.	Electrical elements must be inspected regularly for safety; be careful not to overheat with the heat source; they can dig and burrow in substrate.
Veiled Chameleon (*Chamaeleo calyptratus*)	A rotating habitat, live feeding food items, and training can be enriching	Be careful the chameleon does not get wrapped up or stuck in an over-enriched environment.

### 6.6. Institutional Support

As mentioned earlier, because parent-reared owls that come through rehabilitation facilities are such poor subjects for participation in ambassador animal programs, zoo leaders should be aware of the challenges with these birds and avoid acquiring them from rehabilitation centers. In zoos that already have parent-reared owls in their programs, which are not participating voluntarily, zoo leaders should commit to reducing the stress and improving the welfare of these birds by reexamining all current practices, up to and including the consideration of constructing an exhibit in which the owl can spend the rest of its life.

Zoo programs should also commit to supporting daily observations of behavior and a yearly diagnostic evaluation to ensure the animal’s overall health and welfare. Animals that were injured in the wild should be checked closely each year, or more often, to ensure that the injuries that made the animal non-releasable are not contributing to an overall reduction in welfare as it progresses through life. These welfare assessments and quality-of-life assessments are an essential part of an ambassador animal’s life in human care.

### 6.7. Species-Appropriate Behavior in Programs (Table 9, Table 10 and Table 11)

Many ambassador animal programs feature animals sitting on a gloved hand or being restrained in some way for the length of the presentation. What these programs miss are the most engaging and amazing features of ambassador animals; their species-appropriate behavior. Studies have shown that active animals attract people [69], and that visitor perceptions of animals improve with increased interactions with animals [70]. 

When an ambassador animal program has expert trainers with a good working knowledge of positive reinforcement training techniques, the stage is set for an engaging demonstration of behavior that employs an animal’s natural behavior as a vehicle for educational messages. Effective ambassador animal programming fosters deep thought and can spur conservation action. The following tables offer suggestions for species-appropriate behavior that can be added to ambassador animal programs.

**Table 9:** Birds commonly used in ambassador animal programs and examples of species-appropriate behavior [61].

Bird	Species-Appropriate Behavior
Red-tailed Hawk (*Buteo jamaicensis*)	Flying from perch to perch with experienced trainers; stepping out of crate and going to a tree; voluntarily stepping up onto a glove or branch; eating in front of guests.
Laughing Kookaburra (*Dacelo novaeguineae*)	Vocalization on cue; flying from perch to perch for experienced trainers; slamming a rubber snake or lizard on cue.
Tawny Frogmouth (*Podargus strigoides*)	Cryptic posture on tree branch on cue; voluntarily stepping onto a presenter’s hand and onto a perch; eating during presentation.
Blue and Yellow Macaw (*Ara ararauna*)	Voluntarily stepping out of a transport unit onto trainer’s hand; vocalizing on cue; chewing off a large piece of wood from a 2 × 4 to show their strength; flying on cue for experienced trainers.

**Table 10:** Mammals commonly used in ambassador animal programs and examples of species appropriate behavior [61].

Mammal	Species-Appropriate Behavior
Striped Skunk (*Mephitis mephitis*)	Voluntary crating and stepping onto the hand of presenter; trainer hiding a special treat in a toilet paper roll and mixing with other rolls for the skunk to locate by smell; digging behavior, training to sit on back legs or walk on back legs.
Nine-banded Armadillo (*Dasypus novemcinctus*)	Training to voluntarily go into and out of crate; digging in mulch for treats; being put inside round pen for guests to stand or sit near.
African-crested Porcupine (*Hystrix cristata*)	Training to walk next to a trainer; training to crate on cue; foraging and digging in mulch; climbing to the top of stump and sitting for duration.
Virginia Opossum (*Didelphis virginiana*)	Training to voluntarily crate and come out to presentation area; moving from one trainer to the other; climbing on stump or perch; digging; foraging for food items; being trained to pick up newspaper pieces and carry them in tail like they do with nesting material.

**Table 11:** Reptiles commonly used in ambassador animal programs and examples of species-appropriate behavior [61].

Reptile	Species-Appropriate Behavior
African Spurred Tortoise (*Geochelone sulcata*)	Voluntary crating when small; walking from one trainer to another on cue; walking over obstacles like small tree branches or mulch; feeding from trainer; letting guests feed browse held a safe distance from tortoise’s mouth.
Ball Python (*Python regius*)	Training to voluntarily go into travel crates or onto a trainers hand; using peg boards for snakes to explore; setting tubes and boxes with a hole in them for the snake to investigate; snakes can be taught to follow a scented trail made by dragging a dead mouse or other food item.
Savannah Monitor (*Varanus exanthematicus*)	Training to voluntarily walk to presentation site or walk out of transport carrier; can be trained to walk on a harness and go for walks; teaching the monitor to climb on stump or branches; teaching the monitor to use its sense of smell to locate hidden food.
Veiled Chameleon (*Chamaeleo calyptratus*)	Training to voluntarily go into crate or onto a presenters hand or tree branch; letting guests hold a cricket for chameleon to snatch and eat; teaching to move to and from various branches on a small presentation tree.

## 7. Continued Evaluation

No single metric can be used to provide an overall measure of welfare [8]. However, we can design enrichment and husbandry programs based on desirable behavioral outcomes, which can lead us to establish inputs that create desirable experiences [56]. Through the careful evaluation of observable behavior and re-structuring our training and enrichment programs based on observable behavior, we can pursue our welfare goals.

To ensure that improvements in ambassador animal welfare evolve and grow, training and welfare information must be available to those who manage ambassador programs and are in a position to support change. Multiple aspects of ambassador animal programs must be critically evaluated, such as the following in Table 12 below:

## 8. Conclusions

Ambassador animals are the only animals in most zoological facilities for whom restraint and handling are a daily part of their lives. As such, they are more likely to be exposed to aversive human interactions that are not as prevalent in exhibit-only animals with protected contact. The potential for reduced welfare exists where programmatic pressures influence animal handling strategies in negative ways. Caregivers may rush to meet timelines, struggle to keep animals in place during programs, or lose sight of an animal’s wellbeing when cornering and grabbing it, since forcefully moving the animal is quicker and easier than teaching it to voluntarily participate in loading for transport. Although the handling and training of ambassador animals has significantly improved over the years, there is still a considerable gap between the current state of ambassador animal welfare and a future where ambassador animals receive equal or greater levels of consideration compared to their exhibit-only zoo and aquarium counterparts when it comes to animal handling and welfare.

There is a need to ensure welfare is not just a box that is checked on the daily records of an ambassador animal. Improving welfare should be a routine part of daily activities and institutional culture, rather than an inconvenience that hinders an ambassador animal program’s performance. Through nurturing and supportive staff training and mentoring, institutions can inspire a commitment to choosing what is right over what is convenient. A forward-thinking ambassador animal program will identify strategies that can create a culture of ongoing evaluation and continuous improvements to obtain optimal animal welfare.

## Figures and Tables

**Table 1:** Animal training terms with definitions and examples, as used in this paper.

Term	Definition	Example
Control	The ability to produce desired results predictably and effectively in a given situation [12].	An animal moves toward a crate and the trainer opens the door for the animal to go inside and receive the food reinforcer.
Choice	The presence of multiple, relatively salient discriminative stimuli, at least one of which is an S^D^, i.e., cue [17].	The trainer gives a cue for the animal to go to a stump but the animal goes to a warm, sunny rock instead.
Positive reinforcement	A contingency that involves the presentation of an event or a stimulus following a behavior that increases the rate of response [26].	A trainer provides a valued food item when an animal steps onto a station to increase the likelihood it will step on the station in the future.
Negative reinforcement	A reinforcement procedure or experience in which a behavior is followed by the removal of, or a decrease in the intensity of, an aversive stimulus [26].	To increase the likelihood a serval cat will go into a crate, a trainer puts a small bit of pressure on the cat’s rear end. The cat moves away from the aversive stimulus towards the crate.
Positive punishment	Procedure that involves the presentation of an event or stimulus following a behavior with the effect of decreasing the response rate [26].	An animal tugs ahead on a leash and the trainer yanks back on it, causing the animal’s tugging behavior to decrease in the future.
Extinction	The breaking of the contingency between an operant behavior and its consequence [26].	An animal learning to go into a crate for food reinforcers finds that food reinforcers are withheld and are no longer available.
Learned helplessness	Exposing an animal to inescapable aversive stimuli—eventually the animal gives up trying to escape, even when escape is made easily achievable [12,26].	An animal struggling to get away from a handler finally stops struggling and remains calm, even after the restraint is removed and escape is available.
Stressor	Any external stimulus (chemical, biological, environmental) that induces a stress response in an organism [27].	A veterinarian with a history of aversive interactions with an animal enters the area near the animal, and the animal exhibits avoidance behavior.
Stress	In the general adaptation model, a stress reaction is the neuroendocrine responses to a stressor, whether it is a positive or negative experience for the animal [27,28].	An increase in glucocorticoid levels is noted when a female guinea pig is moved from being housed on exhibit to an off-exhibit holding area.
Distress	When an animal is unable to adapt to chronic or acute stressors [27,29].	Increased fecal corticoid levels in clouded leopards are noted over time when housed in a setting without ideal husbandry practices.
Eustress	“Good stress” plays a role in assessing and disposing of stressors, and allowing the individual to prepare for and survive future challenges [27].	An ambassador animal sees a guide dog enter the theater. Stress is the first response; then, as the dog walks away or lays down, the stress is relieved.
Intrusiveness	The degree of counter-control, choice, and consent for the learner [30].	Ignoring an animal’s escape behavior while moving to pick it up.

**Table 2:** Examples of minimal criteria to consider prior to acquisition for ambassador animal program.

Animal	Health Status/ Behavior	Justification for Inclusion in AA Program	Housing and Transport Considerations	Assessment
Great Horned Owl	Adult-rehabilitated w/wing injury. Not calm around people. Expert-level handling only.	Need another owl because currently held owl is no longer suitable.	8′ × 10′ cage in AA holding, 400 size vari kennel.	Not a good candidate for AA program because of escape behavior and medical condition–deny acquisition.
Great Horned Owl	Six months old, hand-raised bird. Readily approaches people. Easy to handle for education staff at previous facility.	Good guest attraction, easy to handle, and may free-fly in programs if training is achieved.	12′ × 20′ outdoor flight aviary at ambassador building. 400 size vari kennel.	Good addition because it is comfortable w/humans and the opportunity for important messaging. Approve acquisition.
North American Opossum	Hand-raised in rehab facility. Very tame; approaches people willingly. Easy to handle.	We lack mammals in program and want to represent the relationship between opossums and humans.	6′ × 10′ cage in small mammal holding. 300 size vari kennel.	Likely a good candidate for AA program due to its comfort with people and handling. Approve acquisition.
Hedgehog	Has chronic respiratory infection. Has been a voluntary participant in programs at previous zoo.	Fits our program plan for small mammals and easy to transport.	3′ × 4′ cage in AA holding. Transport in 100 size vari kennel.	Medical status not acceptable to veterinarians. Deny acquisition.

**Table 12:** Questions to ask about ambassador animal management and staff administration of animal ambassador programs.

Category	Question
Housing	Is housing optimal and variable during transport, during programs, and when not part of a program (permanent housing)?
Staff	Are staff aware of the behavioral cues that animals provide in response to trainer actions and are the conditions they create adequate to meet the institutional welfare goals? Is staff training resulting in effective animal behavior outcomes? Are staff members working with ambassador animals evaluated and promoted according to their observable skills instead of their title or years of service? Does the ambassador animal team exhibit trusting and supportive relationships?
Institutional Culture	Does the institutional culture support staff effectiveness and optimal animal welfare outcomes? Do the institutional culture and staff actions support flexible and adaptable management to achieve optimal animal welfare? Does the institutional culture support research into strategies for optimizing ambassador animal welfare?
Animal Needs	Are ambassador animals’ nutritional needs being met and overseen to ensure that excessive or inappropriate feeding does not occur? Are animals gently handled during transport and during programs, or is there unnecessary jostling? Are ambassadors provided with effective environmental enrichments using the same expectations that exist for all display animals? Are animals’ social needs appropriately addressed throughout all life stages and during routine housing?
Management	Are the “right” species and individuals being used in ambassador programs? Is funding for ambassador animals sufficient to meet animal welfare needs? Are data being used to drive decision-making surrounding the management of ambassador animals? Are animal training methods evaluated with evidence-based protocols?

## Data Availability

Data are contained within the article.
